# COVID-19 and non–COVID-19 pneumonia: a comparison

**DOI:** 10.1080/07853890.2021.2010797

**Published:** 2021-12-02

**Authors:** Chiara Di Mitri, Giuseppe Arcoleo, Emilia Mazzuca, Gaetana Camarda, Enzo Massimo Farinella, Maurizio Soresi, Antonio Carroccio, on behalf of the IMUSG

**Affiliations:** a Internal Medicine Unit, V. Cervello Hospital, Palermo, Italy; b Pneumology Unit, V. Cervello Hospital, Palermo, Italy; c Infectious Diseases Unit, V. Cervello Hospital, Palermo, Italy; d Internal Medicine, PROMISE Department, University of Palermo, Palermo, Italy

**Keywords:** COVID-19, pneumonia, SARS-CoV-2, mortality, elderly, HRCT, ground-glass opacities, resources, health system, hospitalization

## Abstract

**Background:**

The COVID-19 pandemic has caused the relocation of huge financial resources to departments dedicated to infected patients, at the expense of those suffering from other pathologies.

**Aim:**

To compare clinical features and outcomes in COVID-19 pneumonia and non-COVID-19 pneumonia patients.

**Patients and methods:**

53 patients (35 males, mean age 61.5 years) with COVID-19 pneumonia and 50 patients (32 males, mean age 72.7 years) with non-COVID-19 pneumonia, consecutively admitted between March and May 2020 were included. Clinical, laboratory and radiological data at admission were analyzed. Duration of hospitalization and mortality rates were evaluated.

**Results:**

Among the non-COVID patients, mean age, presence of comorbidities (neurological diseases, chronic kidney disease and chronic obstructive pulmonary disease), Charlson Comorbidity Index and risk factors (tobacco use and protracted length of stay in geriatric healthcare facilities) were higher than in COVID patients. The non-COVID-19 pneumonia group showed a higher (24% vs. 17%), although not statistically significant in-hospital mortality rate; the average duration of hospitalization was longer for COVID patients (30 vs. 9 days, *p* = .0001).

**Conclusions:**

In the early stages of the COVID pandemic, our centre noted no statistical difference in unadjusted in-hospital mortality between COVID and non-COVID patients. Non-COVID patients had higher Charlson Comorbidity Scores, reflecting a greater disease burden in this population.

**Key Messages**
- In March 2020, the COVID-19 disease was declared a pandemic, with enormous consequences for the organization of health systems and in terms of human lives; this has caused the relocation of huge financial resources to departments dedicated to infected patients, at the expense of those suffering from other pathologies.
- Few published reports have compared COVID-19 and non-COVID-19 pneumonia. In our study, performed in a geographic area with a low prevalence of SARS-CoV-2 infection, we found few statistically significant differences in terms of clinical characteristics between the two groups analyzed.
- In the early stages of the COVID pandemic, our centre noted no statistical difference in unadjusted in-hospital mortality between COVID and non-COVID patients. Non-COVID patients had higher Charlson Comorbidity Scores, reflecting a greater disease burden in this population

## Introduction

Since March 2020, the COVID-19 pandemic has enormously affected the lifestyle and healthcare systems of every country, causing the relocation of huge financial resources to departments set aside for infected patients. This has often happened at the expense of patients with other pathologies. Indeed, entire hospitals have been transformed into COVID-19 health centres, leading to a reduction in admissions for heart attacks and strokes, with a consequent increased mortality rate for cardiovascular diseases [1–3].

Furthermore, there have been reports of a decrease in outpatients being followed up for chronic diseases, a significant reduction in cancer screening [4] and the deprioritization of elective surgery in many cases, to preserve hospital capacity for COVID-19 patients [5].

The impact of the pandemic on Southern Italy during the first months of 2020 was quite different from the situation in Northern Italy, as many southern regions were considered to be at low levels of transmission and no substantial increase in deaths from COVID-19 was observed. Nevertheless, even in these regions, a considerable part of the professional, structural and economic resources of public health services has been redirected to face the COVID-19 emergency. The cost-effectiveness of this choice has been difficult to evaluate, therefore the data analysis of clinical outcomes in COVID-19 and non-COVID-19 patients would be of invaluable help when making future decisions.

The aim of the present study was to compare the clinical features and outcomes of COVID-19 and non-COVID-19 patients consecutively hospitalized for pneumonia between March and May 2020, in different wards of the “V. Cervello” Hospital in Palermo, Sicily, Italy.

## Materials and methods

Our retrospective study was conducted on the medical records of 103 patients, consecutively admitted for pneumonia to the “V. Cervello” Hospital in Palermo from mid-March to the end of May 2020. 53 were admitted to the “Infectious Diseases COVID- 19 Unit” and to the “Pneumology COVID-19 Unit” with a confirmed diagnosis of COVID-19 pneumonia; the remaining 50 were admitted to the “Internal Medicine Unit” (non-COVID-19 Unit).

COVID-19 infection was diagnosed by a positive SARS-CoV-2 Real Time-Polymerase Chain Reaction (RT-PCR) on a nasopharyngeal swab. Non-COVID-19 patients were defined by at least two negative SARS-CoV-2 RT-PCRs on nasopharyngeal swabs taken 72 h apart.

Inclusion criteria were: a) evidence of pneumonia at High Resolution Computed Tomography (HRCT) scan, b) availability of the complete laboratory data and a detailed clinical history, c) availability of complete information about the outcome (discharge/death) and duration of hospitalization, d) results of at least two assays for SARS-CoV-2 RT-PCR on a nasopharyngeal swab.

Exclusion criteria were: a) age < 18 years; b) incomplete clinical records, lack of laboratory or imaging data.

All the symptoms and comorbidities observed were defined according to the current guidelines. Appendix 1 summarizes the definitions used in the study. The Charlson Comorbidity Index (CCI) was used to standardize comorbidity distribution between COVID and non-COVID patients.

For each patient, data including complete blood cell count, C reactive protein (CRP), lactate dehydrogenase (LDH), D-dimer, ferritin, INR, fibrinogen, procalcitonin, serum creatinine and brain natriuretic peptide (BNP) were collected. All the analyses were performed at the Central Laboratory of the Hospital, using commercial kits.

HRCT imaging findings were classified by radiologists unaware of the SARS-CoV-2 RT- PCR results as ground-glass opacities (GGO), pulmonary consolidations or a mixed pattern.

Specimens from bronchoalveolar lavage (BAL) or sputum were collected for microbiological culture in a percentage of the patients. Culture tests on respiratory samples were performed within the first three to five days of hospitalization, in the case of lack of response to the empirical antibiotic treatment already commenced in the Emergency Department.

Serum assays for *Chlamydophila pneumoniae* and *Mycoplasma pneumoniae*, Quantiferon for Mycobacterium tuberculosis (MTB), *Legionella pneumophila* and *Streptococcus pneumoniae* urinary antigens and RT-PCR for MTB and other respiratory viruses (e.g. influenza virus) by nasal swabs were also performed according to the previously described indications.

### Statistical analysis

The statistical analysis was performed with SPSS 21 and GraphPad Prism 6.0 for Windows.

Data were expressed as means ± standard deviations (SD) for the parameters with a Gaussian distribution and as medians and range for the parameters with a non-Gaussian distribution. Comparisons were made using parametric (Student’s) or non-parametric (Mann–Whitney U) tests, where appropriate. *p-*values ​​≤ .05 were considered statistically significant.

The study was registered on Clinicaltrials.gov (registration number: NCT 04507893), accessible at: https://www.clinicaltrials.gov/ct2/show/NCT04507893?cond=COVID-19&cntry=IT&city=Palermo&draw=2&rank=3 and was approved by the Ethics Committee of the University Hospital of Palermo (n. 9/2020).

## Results

### Patient characteristics

A total of 107 patients with a diagnosis of pneumonia were hospitalized in the three Units taking part in the study, during the period evaluated. Of these patients, only four were excluded because of incomplete clinical records. The data of the other 103 patients were included. Table 1 shows the demographic and clinical features of the study groups. The mean age of the subjects in non-COVID-19 pneumonia (NCP) group was higher than in the COVID-19 pneumonia group (mean in years 72.7 vs. 61.5; *p* = .0001).

**Table 1.** Demographic and clinical features of non-COVID-19 pneumonia and COVID-19 pneumonia patients.

	NCP controls (non-COVID-19) *N* = 50	Cases (COVID-19) *N* = 53	*p*-value
Demographic characteristics			
Age (mean ± SD) in years	72.7 ± 12.4	61.5 ± 16.6	**.0001**
Male sex: number (%)	32 (64)	35 (66.7)	.83
BMI (mean ± SD) in kg/m^2^	27.3 ± 8.8	26.5 ± 4.4	.55
Symptoms: number (%)			
Fever	42 (84)	47 (88)	.303
Respiratory symptoms	39 (78)	39 (73)	.560
Non-respiratory symptoms	10 (20)	10 (19)	.636
Coexisting conditions — number (%)			
Hypertension	21 (42)	28 (53)	1
Diabetes	13 (26)	8 (15)	1
Neurological diseases	18 (36)	9 (17)	**.05**
Ischaemic Cardiomyopathy	11 (22)	10 (18)	1
CKD	10 (20)	3 (5.6)	**.05**
COPD	14 (28)	7 (13)	**.05**
Asthma	3 (6)	2 (3.7)	.672
OSAS	1 (2)	2 (3.7)	1
Cancer	5 (10)	2 (3.7)	1
Median CCI (IQR)	5 (0–10)	2 (0–9)	**.0001**
Risk factors: number (%)			
Obesity	16 (32)	9 (17)	.101
Current tobacco use	14 (28)	4 (7.5)	**.03**
Geriatric healthcare facilities	15 (30)	4 (7.5)	**.005**
Respiratory conditions			
Respiratory failure at admission: number (%)	27 (54)	35 (67)	.23
O_2_-supplementation at admission L/min (IQR)	2 (0–15)	2 (0–25)	.951
NIV: number (%)	1 (2)	10 (19)	**.0001**
Orotracheal intubation	0 (0)	2 (4)	.49

Abbreviations: CCI: Charlson Comorbidity Index; IQR: denotes interquartile range; NIV: non-invasive ventilation; SD: standard deviation. The numbers in bold indicate the statistically significant values of *p*-value.

No differences in clinical symptoms were found between the groups. Patients with NCP showed a significantly higher frequency of coexisting neurological diseases (36% vs. 17%), chronic kidney disease (CKD) (20% vs. 5.6%) and chronic obstructive pulmonary disease (COPD) (28% vs. 13%) (*p* = .05, for all).

The Charlson Comorbidity Index (CCI) was higher in NCP than in SARS-CoV-2 pneumonia patients (controls: median 5 vs. cases: median 2; *p* = .0001).

Among the risk factors, current tobacco use (28% vs. 7.5%; *p* = .03) and direct admission from Geriatric Healthcare Facilities (Health Residences for non-self-sufficient people usually with chronic diseases) (30% vs. 7.5%; *p* = .005) were more frequent in the NCP than in the COVID-19 patients.

The use of non-invasive ventilation (NIV) during hospitalization was statistically more frequent in the COVID-19 pneumonia group (2% vs. 19%; *p* = .0001). Orotracheal intubation was required for only two patients with COVID-19 pneumonia (4%), who were transferred to an ICU.

#### Laboratory tests

Several statistically significant differences were observed between the two study groups as regards the haemato-chemical parameters: NCP patients had higher values of white blood cell count (WBC) (median 13,990 vs. 7420/mm^3^; *p* = .0001), neutrophil count (median 11,164 vs. 5535/mm^3^; *p* = .0001), D-dimer (median 705 vs. 417 ng/ml; *p* = .03), procalcitonin (median 0.28 vs. 0.1 ng/mL; *p* = .05) and BNP (median 189 vs. 47 ng/mL; *p* = .002) than COVID-19 pneumonia patients.

LDH was, however, higher in COVID-19 pneumonia patients (median 309 vs. 218 mU/mL; *p* = .02) (see Appendix 2).

#### HRCT pattern

**Figure 1.**
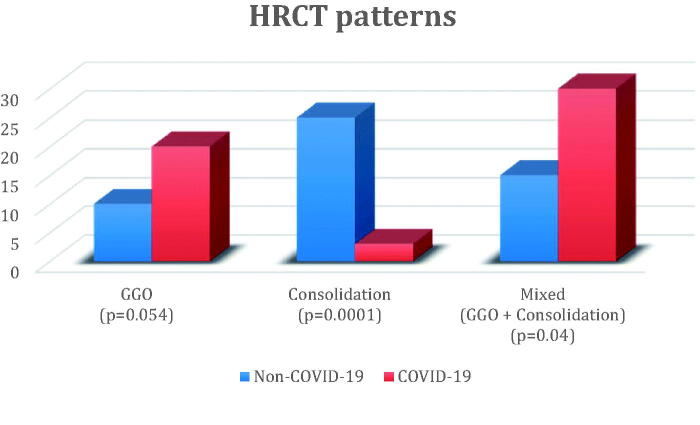
HRCT patterns in patients with non-COVID-19 and COVID-19 pneumonia. Abbreviations: HRCT: high resolution chest tomography, GGO: ground glass opacity, *p*: *p*-value

The HRCT scans showed various pulmonary patterns: GGO, pulmonary consolidations or a mixed pattern in both groups [6]. Figure 1 shows the HCRT patterns of the study groups.

A statistically significant higher frequency of the frank consolidation pattern was found in NCP patients (48% vs. 5.6%, *p* = .0001), while the mixed-type pattern was more frequent in COVID-19 pneumonia (54.7% vs. 30%, *p* = .04). Bilateral pulmonary involvement was more frequent in COVID-19 patients than in NCP (90.6% vs. 66%, *p* = .01). For details, see Appendix 3.

#### Microbiological tests

The frequency of culture isolations on respiratory samples in NCP and COVID-19 pneumonia was not statistically different. In detail, among the COVID-19 patients, 20 respiratory specimens (BAL or sputum) were tested for microbiological culture and 15 were found positive (75%); among the NCP patients, 16 specimens were tested and 14 found positive (87%).

**Figure 2.**
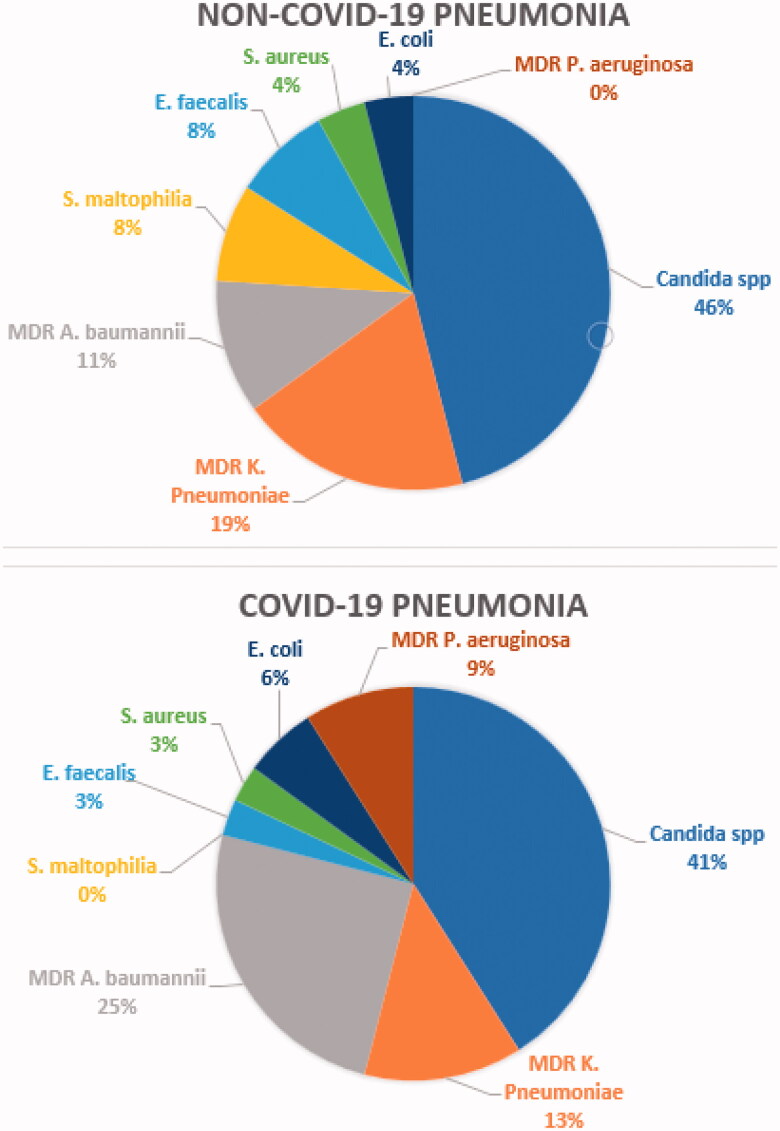
The most common isolations on BAL or sputum culture of non-COVID-19 and COVID-19 patients.

The most common germs isolated in COVID-19 patients were, in order of frequency: *Candida* species, multi-drug resistant (MDR) *Acinetobacter baumannii*, MDR *Klebsiella pneumoniae*, MDR *Pseudomonas aeruginosa*, *Escherichia coli* and, lastly, *Enterococcus faecalis* and *Staphylococcus aureus* (Figure 2).

Furthermore, serum immunoglobulin IgM for *Mycoplasma pneumoniae* tested positive in one patient and Quantiferon for MTB tested positive in another patient.

By contrast, the most frequently isolated germs in the NCP group were: *Candida* species (12 isolates), MDR *Klebsiella pneumoniae* (5 isolates), MDR *Acinetobacter baumannii* (3 isolates), *Stenotrophomonas maltophilia* (2 isolates), *Enterococcus faecalis* (2 isolates), *Staphylococcus aureus* (1 isolate), *Escherichia coli* (1 isolate). In the same group, Coronavirus E229 was detected on a nasopharyngeal swab for respiratory viruses.

No urinary antigens of *Legionella pneumophila* or *Streptococcus pneumoniae* were identified in either of the groups.

#### Mortality and hospitalization

In our study, the NCP group showed a higher, although not statistically significant, in-hospital mortality rate (24% vs. 17%) than the COVID-19 pneumonia group; conversely, the average length of hospitalization was significantly longer in the COVID-19 pneumonia patients (Table 2).

**Table 2.** In-hospital mortality rate and length of hospitalization of non-COVID-19 pneumonia and COVID-19 pneumonia patients.

	NCP controls (non-COVID-19) *N* = 50	Cases (COVID-19) *N* = 53	*p*-value
Deceased: number (%)	12 (24)	9 (17)	.468
Length of hospitalization in days in the whole population (median and range)	9.5 (1–36)	30 (12–80)	**.0001**
Length of hospitalization in days in patients discharged (median and range)	9.0 (1–36)	30 (12–80)	**.0001**
Length of hospitalization in days in patients who died in hospital (median and range)	11.5 (3–27)	25 (14–37)	**.0001**

The numbers in bold indicate the statistically significant values of *p*-value.

Considering the 103 patients as a whole, deceased patients were significantly older (mean in years 79.6 vs. 64.5; *p* = .0001), showed a significantly higher frequency of neurological diseases (48% vs. 21%; *p* = .03) and a higher Charlson Comorbidity Index (CCI) (Deceased: median CCI 5 *vs* discharged: median CCI 3; *p* = .02).

Furthermore, deceased patients had lower values of blood lymphocyte count (median 742 vs. 1105 N/mm^3^, *p* = .01), higher values of CRP (median 14 vs. 5.1 mg/dl, *p* = .0001) and procalcitonin (median 1.05 vs. 0.1 ng/ml, *p* = .0001) and showed a higher frequency of renal (67% vs. 31%) and respiratory failure (81% vs. 55%) at admission (*p* = .05 for both), than the patients who were discharged. Finally, the frequency of microbiological isolation on BAL or sputum was higher in the deceased than in discharged patients (Table 3).

**Table 3.** Demographic, laboratory and clinical features of all discharged and deceased patients included in the study.

	Discharged	Deceased	*p*-value
	*N* = 82	*N* = 21	
Demographical data			
Age in years (mean ± SD)	64.5 ± 15.7	79.6 ± 11.7	**.0001**
Male sex: number (%)	53 (65)	14 (66)	1
Comorbidities and associated risk factors: no. (%)			
Hypertension	41 (50)	8 (38)	.461
Diabetes	17 (21)	4 (19)	.767
Neurological diseases	17 (21)	10 (48)	**.03**
Ischaemic Cardiomyopathy	17 (21)	4 (19)	1
CKD	10 (12)	3 (14)	.488
COPD	17 (21)	4 (19)	1
Cancer	5 (6)	2 (10)	.679
Geriatric healthcare facilities	13 (16)	6 (28)	.21
Median CCI (IQR)	3 (0–10)	5 (2–9)	**.02**
Laboratory tests			
Lymphocyte count (median ± IQR) – N/mm^3^	1105 (294–19,688)	742 (384–2,758)	**.01**
Platelet count (median ± IQR) – N/mm^3^	207,500 (36,000–640,000)	200,000 (55,000–383,000)	.922
PCR (median ± IQR) – mg/dL	5.1 (0.04–27.4)	14 (3.9–31)	**.0001**
LDH (median ± IQR) – mU/mL	243 (23–1,020)	315 (161–527)	.105
BNP (median ± IQR) – ng/mL	72 (10–1,492)	252 (10–714)	.061
PCT (median ± IQR) – ng/mL	0.1 (0.01–64.5)	1.05 (0.07–60)	**.0001**
Other associated factors			
Renal failure at admission: number (%)	25 (31)	14 (67)	**.05**
Respiratory failure at admission: number (%)	45 (55)	17 (81)	**.05**
NIV: number (%)	10 (12)	1 (5)	.45
Culture isolations on respiratory samples (sputum or BAL) (%)	13 (16)	15 (71)	**.0001**

Abbreviations: BNP: brain natriuretic peptide; CCI : Charlson Comorbidity Index; CRP : C reactive protein; IQR : interquartile range; LDH : lactate dehydrogenase; NIV : non-invasive ventilation; PCT: procalcitonin; SD: standard deviation; BAL: bronchoalveolar lavage. The numbers in bold indicate the statistically significant values of *p*-value.

The comparative analysis between deceased and discharged patients in the group with COVID-19 pneumonia alone is shown in Table 4.

**Table 4.** Comparison between discharged and deceased patients with COVID-19 pneumonia.

	Discharged Covid-19	Deceased Covid-19	*p*-value
	*N* = 44	*N* = 9	
Demographic characteristics			
Age (mean ± SD) in years	58.2 ± 15.7	77.3 ± 11.2	**.001**
Male sex: number (%)	28 (64%)	7 (78%)	.47
Coexisting conditions: number (%)			
Hypertension	23 (52%)	5 (55%)	1
Diabetes	7 (16%)	1 (11%)	1
Neurological diseases	7 (16%)	2 (22%)	.663
Ischaemic cardiomyopathy	8 (18%)	2 (22%)	1
Chronic kidney disease	2 (4%)	1 (11%)	0
Chronic obstructive pulmonary disease	4 (9%)	3 (33%)	.08
Cancer	2 (4%)	0 (0%)	.1
Obesity	5 (11%)	4 (44.4%)	**.03**
Geriatric healthcare facilities	3 (7%)	1 (11.1%)	1
Laboratory tests			
Median lymphocyte count (IQR) – N/mm^3^	1198 (461–19,688)	651 (384–1080)	**.002**
Median platelet count (IQR) – N/mm^3^	225,500 (72–640,000)	183,000 (55–288,000)	.176
Median D-dimer (IQR) – ng/mL	398 (65–8971)	845 (150–6722)	.105
Median neutrophil count (IQR) – N/mm^3^	5376 (1885–33,600)	8192 (3784–10,509)	.308
Median BNP (IQR) – ng/mL	29 (10–1,492)	168 (10–679)	.408
Median CRP value (IQR) – mg/dL	4.6 (0.1–27.1)	15.5 (3.9–27.4)	**.003**
Median LDH (IQR) – mU/mL	301 (136–1,020)	440 (180–527)	**.05**
Median procalcitonin (IQR) – ng/mL	0.08 (0.01–15.2)	1.05 (0.7–10.2)	**.02**
Other associated conditions			
Respiratory failure at admission: number (%)	28 (64%)	7 (78%)	.47
NIV during hospitalization: number (%)	9 (20%)	1 (11%)	.67
Kidney injury at admission: number (%)	12 (27%)	5 (56%)	.113
Culture isolations on respiratory samples (%)	10 (23%)	5 (56%)	.1

Abbreviations: BNP: brain natriuretic peptide; CRP: C reactive protein; IQR: interquartile range; LDH: lactate dehydrogenase; NIV: non-invasive ventilation; SD: standard deviation; WBC: white blood cells. The numbers in bold indicate the statistically significant values of *p*-value.

Table 5 summarizes some parameters regarding the resource allocation during the study period. We included four Units which historically received patients suffering from pneumonia: Pneumology, Infectious Diseases, Internal Medicine and Intensive Care Units. Three of these units were converted in “COVID Units” since 1 March 2020: Pneumology, Infectious Diseases and Intensive Care. After 1 March 2020, there was a great increase of beds and personnel assigned to the COVID Units, with new beds activated. On the contrary, beds and personnel assigned to the Internal Medicine Unit, the only which still received patients with non-COVID pneumonia, were reduced.

**Table 5.** Bed numbers and staff employed in COVID and non-COVID wards, before and after 1 March 2020.

	Before February	From 1 March 2020 to 31 May 2020	Difference
	28th 2020		(number and %)
Beds in non-COVID Units	41	14	–27 (–65%)
Beds in COVID Units	12	95	+83 (+790%)
Staff (physicians and nurses) employed in COVID Units	34	186	+152 (+547%)
Staff (physicians and nurses) employed in non-COVID Units	158	36	–122 (–77%)

We included in the analysis four Units which historically receive patients with pneumonia: pneumology, infectious diseases, internal medicine and intensive care.

## Discussion

Few published reports have compared COVID-19 and non-COVID-19 pneumonia. In our study, we found few statistically significant differences in terms of clinical characteristics between the two groups analyzed.

From March 2020, SARS-CoV-2 infection spread rapidly throughout Italy, and it became necessary to dedicate more and more health resources to the management of the pandemic.

There was a difference in the spread of the infection between Northern and Southern Italy. In the South of the country, including our region (Sicily), during the study period (from March to May 2020), the spreading rate of the SARS-CoV-2 infection was low, with a median Rt of 0.47, as was COVID-19 mortality (1.1% of all-cause mortality). The Italian National Institute of Statistics (Istat) reported that 263 COVID-19 positive people died between 1 March and 15 May 2020 in Sicily, a region with 5 million inhabitants. In the same period in 2017, there was a total of 11,230 deaths for all causes in the region, 547 of which were from pneumonia, but it was unspecified whether it was community-acquired (CAP), healthcare-acquired (HCAP) or hospital-acquired pneumonia (HAP) [7]. Nevertheless, parts of departments and some entire hospitals were designated to take care of COVID-19 patients. Whether or not this choice was appropriate in this context is difficult to evaluate.

We compared the clinical characteristics and outcomes of patients hospitalized between March and May 2020 for non-COVID-19 pneumonia (NCP) versus those with COVID-19 pneumonia. In the 50 NCP patients, mean age, presence of comorbidities, risk factors and median neutrophil count was higher than in the 53 patients with COVID-19 pneumonia, a result in agreement with the literature [8–11]. On admission to the Emergency Room, both COVID-19 and NCP patients had similar arterial blood gas analysis parameters and non-significant differences in the need for oxygen therapy. However, the patients with COVID-19 pneumonia required non-invasive ventilation more often during hospitalization, a result also in agreement with the literature [10].

The rate of microbial isolation in sputum or BAL cultures was similar in the NCP and COVID-19 pneumonia groups. In both groups, the most commonly detected microorganisms were Candida species, MDR *Pseudomonas aeruginosa*, MDR *Acinetobacter baumannii*, Gram-negative Enterobacterales.

The absence of a defined temporal protocol of collection of respiratory samples for non-Covid Pneumonia patients as well as the variable length of their stay in the Emergency Department did not allow us to correctly deduce the pathogenetic significance of the microbiological respiratory isolations in this subgroup, although the multi-drug-resistance of these microorganisms makes it likely that they were nosocomial superinfections.

We cannot affirm that *Candida* isolation represented a super-infection in either of the groups, as it often reflects simple colonization. No blood cultures tested positive for *Candida* and this could suggest the likelihood of the second hypothesis. However, considering the often critical clinical conditions of Covid Pneumonia patients, all culture isolations were followed up with a targeted treatment.

In our group of COVID patients, we found a 23% frequency of bacterial superinfections, which is close to other reports in the literature [12,13].

There were no cases of COVID-19 Associated Pulmonary Aspergillosis (CAPA), unlike findings in some cases in the literature [12].

The single positive Quantiferon finding occurred in the COVID Pneumonia group and was not associated with isolation of *Mycobacterium tuberculosis* from the culture of sputum or bronchoalveolar lavage, or with a positive nucleic acid amplification test. It was therefore interpreted as a latent tuberculosis infection [14].

As concerns the frequency of deaths, our study showed a higher, although a not statistically significant percentage of in-hospital mortality in the group of NCP patients compared to COVID-19 (24% vs. 17%): the better outcomes in COVID-19 patients were likely influenced by a lower frequency of comorbidities and younger age, as demonstrated by a lower Charlson Index, in agreement with the current literature [11]. We intended our study to be a snapshot of the “real world” that each clinician faces every day. It shows that in a period and an area of low COVID incidence, the conditions of patients with non-COVID-pneumonia were as severe as in the COVID-patients, even though many financial, structural and human resources were dedicated to the COVID emergency.

Indeed, our data confirm that pneumonia is a severe disease, with a mortality rate between 10% and 30% in patients aged over 65 with CAP [8,15]. Furthermore, the data underline that notwithstanding the COVID-19-related hospitalizations and deaths, the impact of common pneumonia must not be underestimated.

According to the data of the Italian National Institute of Health (ISS) referred on 29 May 2020, the mortality of COVID-19 patients aged between 60 and 69 years (median age of our study group: 65 years) was 10.3% [16], lower than the 17% of our study. Other studies, however, had highlighted that in the first phase of the pandemic in-hospital mortality was about 30%.

As regards the greater length of hospitalization of COVID-19 patients compared to the NCP group (median: 30 days vs. 9.5 days) we must underline that it depended on the discharge criteria followed for these patients as set out in the European Centre for Disease Prevention and Control (ECDC) guidelines in force at the time of the study, which required the confirmation of the patient’s negativization by at least 2 RT-PCR tests on respiratory samples, taken after an interval of 24 h at least 8 days following the onset of symptoms [17].

Obviously, the limitations of our study should be underlined. First, the study is a retrospective one and, for this reason, some patients whose anamnestic data were not complete were excluded; however, the analysis showed that there were few incomplete records (only 4 out of 107). For this reason, and since all the subjects with non-Covid pneumonia were referred to a single centre, the size of the cohort is limited. Second, the study concerns a brief period of the early stages of the pandemic, in which the incidence of infection in our region was also low and therefore not comparable to the emergency conditions of many areas in Northern Italy. Thus, our results may be only partial and do not apply to areas with a high incidence of infection, hospitalization and deaths due to COVID-19. Third, the study is not strictly useful for a real analysis of cost-effectiveness, as it only displays data comparing COVID-19-related and non-COVID-19 pneumonia. Fourth, we had no data about the Charlson Comorbidity Index before the “COVID emergency” and consequently, we did not evaluate whether there was an increased score for non-COVID patients when resources were limited. Fifth, the absence of a defined collection protocol for respiratory microbiological samples did not allow us to safely interpret NCP culture isolations as primary pathogens, superinfections or colonization.

On the other hand, few studies have previously compared clinical outcomes in COVID-19 and non-COVID-19 pneumonia and there are a few data available in the literature. As far as we know, this is one of the few studies using multiple, different parameters to compare the clinical and prognostic features of these two groups.

The higher, although a not statistically significant percentage of in-hospital mortality in the group of NCP patients compared to COVID-19 patients highlights a still little-debated issue: the need to guarantee fair and adequate access to medical care also for non-COVID-19 patients, especially in those areas with a low prevalence of infection. In our study, in fact, we showed that despite there being a period and an area of low COVID incidence, many financial, structural and human resources were dedicated to the COVID emergency and taken away from other wards, including Internal Medicine and Pneumology.

In conclusion, we showed that in areas or periods with a low incidence of SARS-COV2 infection, the outcomes of non-COVID pneumonia can be identical in severity to COVID pneumonia and that this factor should be better evaluated when making decisions on resource allocation.

## Data Availability

The data that support the findings of this study are available on request from the corresponding author (CDM). The data are not publicly available due to their containing information that could compromise the privacy of participants.

## Registration

The study was registered at Clinicaltrials.gov (registration number NCT04507893), accessible at: https://www.clinicaltrials.gov/ct2/show/NCT04507893?cond=COVID-19&cntry=IT&city=Palermo&draw=2&rank=3

## Appendix 1

### Definitions and diagnostic criteria

Fever was defined as an axillary body temperature higher than 37.5 degrees Celsius.

Hypertension was defined as blood pressure values equal to or higher than 140/90 mmHg [18].

Chronic kidney disease (CKD) was defined as eGFR values lower than 60 ml/min for at least three months: KDIGO 2012 (Kidney Disease: Improving Global Outcomes) guidelines [19].

Diabetes mellitus was defined as fasting glycemic values equal to or higher than 126 mg/dl or glycated haemoglobin higher than 6.5%: ADA 2020 (American Diabetes Association) guidelines [20].

Ischaemic and Hypertensive Heart Disease were defined as a history of myocardial infarction, stable or unstable angina pectoris and the presence of echocardiographic signs of left ventricular hypertrophy associated with a history of hypertension [18,21].

Chronic Obstructive Pulmonary Disease (COPD), defined as the presence of dyspnoea, chronic cough or sputum, associated or not with exposure to risk factors, with signs of persistent bronchial obstruction on spirometry: GOLD 2019 guidelines [22].

Bronchial asthma was defined as a history of coughing, exhalation wheezing and dyspnoea associated with both a documented bronchial obstruction and an excessive variability of this in different functional tests - GINASMA 2019 guidelines [23].

Obstructive Sleep Apnoea Syndrome (OSAS) was defined as the presence of an Apnoea–Hypopnea Index (AHI) equal to or greater than 5 events/hour on polysomnography in the presence of compatible symptoms, or an AHI greater than 15 in the absence of symptoms - AASM criteria [24].

Obesity was defined as Body Mass Index (BMI) values ​​greater than 30 kg/m^2^ [25].

Smoking habit was considered as active smoking or cessation within 6 months before admission.

Respiratory symptoms included: dyspnoea, cough, sore throat, anosmia, rhinorrhea.

Respiratory failure at entry was defined as PaO_2_ values ​​below 60 mmHg in ambient air or the need for FiO_2_ greater than 21% to maintain PaO_2_ values ​​equal to or greater than 60 mmHg or SO_2_ values ​​equal to or greater than 90% when blood gas analytical data at entry were not available [26].

Under neurological diseases, we included both the outcomes of acute cerebrovascular events [27] and chronic neurodegenerative diseases such as Alzheimer’s [28] or Parkinson’s [29] as well as psychiatric diseases such as schizophrenia [30].

Neoplasms considered as comorbidities were both those involving solid organs and the myelolymphatic system.

Multidrug resistance (MDR) was defined as an acquired non-susceptibility to at least one agent in three or more antimicrobial categories [31].

## Appendix 2

### Comparison of laboratory data of non-COVID-19 and COVID-19 pneumonia patients

**Table:**

	Non-COVID-19 patients *N* = 50	COVID-19 patients *N* = 53	*p*-value
Laboratory			
Median WBC count (IQR) – N/mm^3^	13,990 (5200–32,000)	7420 (3890–42,800)	**.0001**
Median lymphocyte count (IQR) – N/mm^3^	929 (294–3,990)	1011 (384–19,688)	.522
Median neutrophil count (IQR) – N/mm^3^	11,164 (3432–30,080)	5535 (1885–33,600)	**.0001**
Median platelet count (IQR) – N/mm^3^	203,000 (36,000–454,000)	208,000 (55,000–640,000)	.518
Median CRP value (IQR) – mg/dL	8.5 (0.04–31)	6.5 (0.1–27.4)	.514
Median LDH (IQR) – mU/mL	218 (23–770)	309 (136–1,020)	**.02**
Median D-dimer (IQR) – ng/mL	705 (135–3,334)	417 (65–6,428)	**.03**
Median ferritin (IQR) – ng/mL	389 (13–3,578)	647 (15–6,428)	.223
Median INR (IQR)	1.19 (0.9–3.4)	1.15 (0.9–12)	.478
Median fibrinogen (IQR) – mg/dL	494 (179–1,394)	538 (130–1,398)	.535
Median procalcitonin (IQR) – ng/mL	0.28 (0.02–64.5)	0.1 (0.01–15.2)	**.05**
Median creatinine (IQR) – mg/dL	1.19 (0.51–10)	1.04 (0.43–10.1)	.222
Median BNP (IQR) – ng/mL	189 (13–920)	47 (10–1,492)	**.002**
Arterial blood gas test			
Median pH (IQR)	7.45 (7.23–7.5)	7.41 (7.32–7.51)	.142
Median PaCO_2_ (IQR) – mmHg	36 (23–90)	38.1 (27–66)	.137
Median PaO_2_ (IQR) – mmHg	61.5 (44–151)	73 (52–149)	.146
Median HCO_3_^–^ (IQR) – mmol/L	25 (15–41)	24.7 (17–38)	.728

Abbreviations: BNP: denotes brain natriuretic peptide; CRP: C reactive protein; IQR: interquartile range; LDH: lactate dehydrogenase; *N*: number; WBC: white blood cells. The numbers in bold indicate the statistically significant values of *p*-value.

## Appendix 3

### Comparison of HRCT pulmonary patterns in non-COVID-19 and COVID-19 pneumonia patients

**Table:**

	Non-COVID-19 patients *N* = 50	COVID-19 patients *N* = 53	*p*-value
HRCT pattern: no. (%)			
Negative CT	1 (2)	1 (1.9)	1
GGO	10 (20)	20 (37.7)	.054
Consolidation	24 (48)	3 (5.6)	**.0001**
GGO + consolidation	15 (30)	29 (54.7)	**.04**
*ρ*=ns			
HRCT distribution: no. (%)			
Negative CT	1 (2)	1 (1.9)	1
Monolateral	16 (32)	4 (7.5)	**.005**
Bilateral	33 (66)	48 (90.6)	**.01**
*ρ* = 0.3, *p* < .01			
Effusion – no. (%)			
Pleural	23 (46)	14 (26)	**.05**
Pericardial	8 (16)	6 (11)	0.57

Abbreviations: CT: denotes computed tomography; GGO: ground-glass opacities; HRCT: high resolution computed tomography; no.: numbers; ns: non-significant; *ρ*: Spearman’s rank correlation coefficient. The numbers in bold indicate the statistically significant values of *p*-value.

## Abbreviations

BAL: Bronchoalveolar lavage
BMI: Body Mass Index
BNP: Brain natriuretic peptide
CCI: Charlson Comorbidity Index
CKD: Chronic kidney disease
COPD: Chronic obstructive pulmonary disease
CRP: C-reactive protein
GGO: Ground glass opacity
HRCT: High resolution computed tomography
LDH: Lactate dehydrogenase
MDR: Multidrug-resistant
MTB: *Mycobacterium tuberculosis*
NCP: non-COVID-19 pneumonia
NIV: Non-invasive ventilation
OSAS: Obstructive sleep apnoea syndrome
RT-PCR: Real time-polymerase chain reaction
WBC: White blood cell count
